# Zika virus replication in glioblastoma cells: electron microscopic tomography shows 3D arrangement of endoplasmic reticulum, replication organelles, and viral ribonucleoproteins

**DOI:** 10.1007/s00418-021-02028-2

**Published:** 2021-09-12

**Authors:** Johannes Wieland, Stefan Frey, Ulrich Rupp, Sandra Essbauer, Rüdiger Groß, Jan Münch, Paul Walther

**Affiliations:** 1 Central Facility for Electron Microscopy, Ulm University, 89081 Ulm, Germany; 2 Bundeswehr Research Institute for Protective Technologies and CBRN Protection, 29633 Munster, Germany; 3 Bundeswehr Institute of Microbiology, 80937 Munich, Germany; 4 Institute of Molecular Virology, Ulm University Medical Center, 89081 Ulm, Germany

**Keywords:** Zika virus, Glioblastoma, Sandfly fever Turkey virus, Electron microscopy, STEM tomography, High-pressure freezing, Replication organelles, Viral ribonucleoprotein

## Abstract

Structural changes of two patient-derived glioblastoma cell lines after Zika virus infection were investigated using scanning transmission electron tomography on high-pressure-frozen, freeze-substituted samples. In Zika-virus-infected cells, Golgi structures were barely visible under an electron microscope, and viral factories appeared. The cytosol outside of the viral factories resembled the cytosol of uninfected cells. The viral factories contained largely deranged endoplasmic reticulum (ER), filled with many so-called replication organelles consisting of a luminal vesicle surrounded by the ER membrane. Viral capsids were observed in the vicinity of the replication organelles (cell line #12537 GB) or in ER cisternae at large distance from the replication organelles (cell line #15747 GB). Near the replication organelles, we observed many about 100-nm-long filaments that may represent viral ribonucleoprotein complexes (RNPs), which consist of the RNA genome and N protein oligomers. In addition, we compared Zika-virus-infected cells with cells infected with a phlebovirus (sandfly fever Turkey virus). Zika virions are formed in the ER, whereas phlebovirus virions are assembled in the Golgi apparatus. Our findings will help to understand the replication cycle in the virus factories and the building of the replication organelles in glioblastoma cells.

**Supplementary Information:**

The online version contains supplementary material available at 10.1007/s00418-021-02028-2.

## Introduction

Zika virus (ZIKV) is an enveloped flavivirus with a positive-sense, single-stranded RNA genome. The virus can be transmitted to humans by the bite of *Aedes* mosquitoes, or from human to human by maternal-to-fetal transmission or, in isolated cases, by sexual transmission (Pierson and Graham 2016). On transmission electron microscopy (TEM), the virions present as spherical particles with a diameter of about 50 nm (Barreto-Vieira et al. 2016; Simmonds et al. 2017). ZIKV is known as an emerging highly pathogenic virus causing severe neurological diseases, but it is also listed among the group of oncolytic viruses (Zhu et al. 2017; Trus et al. 2020; Vanwalscappel et al. 2020). Like other flaviviruses, ZIKV infects glial cells and astrocytes (Li et al. 2018; Protokar et al. 2019; Mustafa et al. 2019). Due to its neurotropism and specificity for glioblastoma stem cells (Zhu et al. 2017), ZIKV is currently considered as an agent for virotherapy of glioblastoma.

The entrance of Zika virions into host cells occurs by virus endocytosis following receptor binding. After endocytosis and vesicle acidification, the approximately 11 kb RNA genome is released into the host cell cytosol. Zika virus infection and replication cause a massive rearrangement of cellular structures (Cortese et al. 2017). Foremost, this results in the formation of an area referred to as viral factory (VF) consisting of largely deranged endoplasmic reticulum that contains luminal vesicles referred to as replication organelles (ROs). Thereby, two membranes separate the lumen of the RO from the cytosol. The outer membrane is part of the ER, and the inner membrane is a luminal ER vesicle. It is usually assumed that these vesicles are formed by invagination of the ER membrane and that they are the site of viral RNA replication because of their optimal milieu for viral RNA synthesis, shielding viral RNA from cytosolic pattern recognition receptors. These structures have already been observed during dengue virus replication (Welsch et al. 2009) and have been called “virus induced vesicles.” In addition, ROs show structural similarities to “double membrane vesicles” (DMVs), ER-derived vesicles with a diameter of approximately 250 nm (Wolff et al. 2020a).

Several studies describe pores that connect the vesicle lumen with the cytosol and could serve as a transport channel to transport the newly synthesized RNA out of the ROs into the cytosol. Welsch et al. (2009) showed tomographic reconstructions of such pores in the “virus induced vesicles” of dengue-virus-infected cells that look like an incomplete membrane fission of the invaginated ER membrane. Cortese et al. (2017); (Fig. 4) also observed pore-like structures in ROs of ZIKV-infected cells. In addition, in the case of coronavirus, recently a very different pore structure has been described: using cryo-TEM tomography, Wolff et al. (2020b) observed molecular pores that span the two membranes of the DMVs. These pores resemble the structure of nuclear pores and are formed by proteins inserted in the two membranes of the ROs. Based on these data, Wolff et al. (2020b) proposed a structural model of viral RNA replication and transfer into the newly formed viral particles (Fig. 4). Through these pores, the viral RNA could be transferred to the cytosol where they could assemble with viral nuclear proteins to ribonucleoprotein complexes (RNPs) that are then mounted into the virions that are newly formed by invagination of the ER. The result is an RNA-loaded virion inside the ER.

We here examined structural changes induced by ZIKV in two patient-derived glioblastoma cell lines and compared them with uninfected control cells. We, thereby, hoped to gain further insight in the replication cycle of ZIKV. To evaluate how ZIKV infection affects the physiological structure of the Golgi apparatus, we compared our samples with a tomogram of sandfly fever Turkey virus, a phlebovirus where the Golgi is the main site of virion formation. Sandfly fever Turkey virus was chosen as a reference to demonstrate how virus infections that utilize the Golgi apparatus for virion formation and transport present on STEM tomography.

For this work, we faced two major methodological challenges: firstly, the gold standard for fixation is rapid freezing from a physiologically defined state, thereby avoiding possible artifacts of immobilization by chemicals (Mielanczyk et al. 2014). For this study, however, we had to immobilize ZIKV-infected samples with glutaraldehyde to fulfill the safety requirements before transport to the freezing device. The second challenge is the electron tomography mode we used. The new gold standard is cryotomography of thin lamellae produced by cryo-focused ion beam (cryo-FIB) milling from frozen hydrated samples (Wolff et al. 2020b). Thereby, all artifacts related to drying of the sample are excluded. In our work, we used freeze-substituted and Epon-embedded samples for tomography. These samples are dehydrated at cold temperatures (−90 °C), which prevents many of the artifacts produced by conventional fixation and dehydration (Walther et al. 2018). The rather stable samples were then analyzed by scanning transmission electron microscopy (STEM) tomography. This approach enables the 3D analysis of larger volumes compared with conventional TEM tomography (Walther et al. 2018; Hohmann-Marriott et.al. 2009). We focused on features of viral replication sites that are difficult to interpret in conventional two-dimensional TEM imaging.

## Materials and methods

### Cell culture

Glioblastoma cell lines were established from fresh tumor tissue and characterized by a panel of cluster of differentiation (CD) antibodies expressed on the surface as well as in the cytoplasm (Sander et al. 2017). Vero B4 cells (ATCC No. CCL-81) were grown in M199 supplemented with 5% fetal calf serum.

### Viruses

For Zika virus infection, the Brazilian strain ZIKV_FB-GWUH-2016 (KU870645) was used, which was isolated from a fetus with microcephaly (Driggers et al. 2016). For infection experiments, the respective cell line was grown to 70% confluency and Zika virus diluted in growth medium was added at a multiplicity of infection of 1. Cells were fixed 48 h after infection. For the phlebovirus infection, Vero B4 90% confluent monolayers were infected with the Turkish virus strain SFTV NC_015411 (Carhan et al. 2010).

### Sample preparation for electron microscopy

Samples were prepared following the protocol of Villinger et al. (2014). Cells were cultivated on carbon-coated sapphire discs with a diameter of 3 mm and a thickness of 160 μm (Engineering Office M. Wohlwend GmbH, Sennwald, Switzerland).

ZIKV-infected cells were prefixed with 2.5% glutaraldehyde with 1% saccharose in 0.1 m phosphate buffer at pH 7.3 for 1 h. Non-infected control cells as well as phlebovirus-infected cells were high-pressure frozen from the medium without prefixation.

Two sapphire discs were clamped together with the cells facing inward. A 50-µm-thick gold ring (diameter 3.05 mm, central bore 2 mm; Plano GmbH, Wetzlar, Germany) located between the discs created enough space for the cells. This setup was then frozen in a Wohlwend HPF Compact 01 high-pressure freezer (Engineering Office M. Wohlwend GmbH, Sennwald, Switzerland).

Samples were freeze-substituted as described in Walther and Ziegler (2002) with a substitution medium consisting of acetone with 0.2% osmium tetroxide, 0.1% uranyl acetate, and 5% water. Over the course of 17 h, the temperature was raised exponentially from −90 °C to 0 °C. Following substitution, the samples were kept at room temperature for 1 h and washed twice with acetone afterwards. The samples were then subsequently embedded in Epon and left at 60 °C for 72 h to allow polymerization.

For TEM, 70-nm-thick sections were cut from the Epon block parallel to the surface of the sapphire disc with a Leica Ultracut UCT ultramicrotome. The slices were then mounted on copper grids.

For STEM tomography, thick sections of 700–900 nm were cut from the Epon block with a 35° diamond knife (diatomeknives.com). A copper grid with parallel bars was glow-discharged with an Edwards plasma cleaning system. The sections were then mounted on the grid, and a droplet of 10% (w/v) poly-l-lysine (Sigma Aldrich) in water was added. The sections were then dried for 5 min at 37 °C. Then, 15 μl of a solution containing 25 nm gold particles (Aurion.com) diluted 1:1 with water was applied to both sides of the sections serving as fiducial markers. Finally, a 5 nm carbon layer was added to each side of the grid using a BAF 300 electron beam evaporation device (opticsbalzers.com).

### Electron microscopy

Conventional TEM imaging was performed with a Jeol JEM 1400 at an accelerating voltage of 120 kV or with a Zeiss TEM 109 at 80 kV. Figure 2 and Supplementary Materials S2 and S3 are composite images recorded as described by Rupp and Ziegler (2019) with a Zeiss Leo 912 Omega TEM (Carl Zeiss AG, Oberkochen, Germany) at 120 kV using the elastically scattered electrons of the zero-loss peak. Digital micrographs were recorded with a 2000 × 2000 pixel camera (TRS, Moorenweis, Germany). Using TRS software, 400 images were recorded in a 20 × 20 raster. The images were then assembled into large composite images.

Tomograms were acquired in STEM mode with a Jeol JEM-2100F at 200 kV accelerating voltage. EM-Tools software (TVIPS, Tietz) was used for the acquisition of the series. A series of bright-field images at tilt angles from −72° to +72° with 1.5° increment was recorded with a Jeol bright-field detector. Illumination time per image was 22 s. Each image had 1024 × 1024 pixels, pixel size was 2.74 nm for 200,000×, 1.38 nm for 400,000×, and 6.85 nm for 80,000×. Ninety-seven images were used for the reconstruction of each tomogram. Phlebovirus-infected cells (Fig. 6) were recorded with a 300 kV field emission STEM (Titan 80–300 TEM, FEI, Eindhoven) using a dark-field detector. For better comparison with the bright-field images, the contrast in Fig. 6b was reversed.

Tomogram reconstruction was done with the programs Etomo and 3dmod (Kremer et al. 1996), which are part of the IMOD software package (vs.4.9.12). Images were aligned and subsequently reconstructed to generate a 3D dataset. The program presets were used, except for the window “Fine Alignment” where under “Global Variables” and “Distortion Solution Type” the option “Full solution” was activated (according to suggestions by Reinhard Rachel). Additionally, in the window “Tomogram Generation,” the option “take logarithm with the densities of” was activated and set to 32,768 (according to suggestion by David Mastronarde on the IMOD list). The segmentations were done with 3dmod from the IMOD package 4.9.12.

## Results

### Structure of uninfected glioblastoma cells

We first studied the effects of glutaraldehyde fixation on cellular structures, since they are altered not only by ZIKV infection but also by the chemical prefixation that was necessary for safety reasons.

**Fig. 1:**
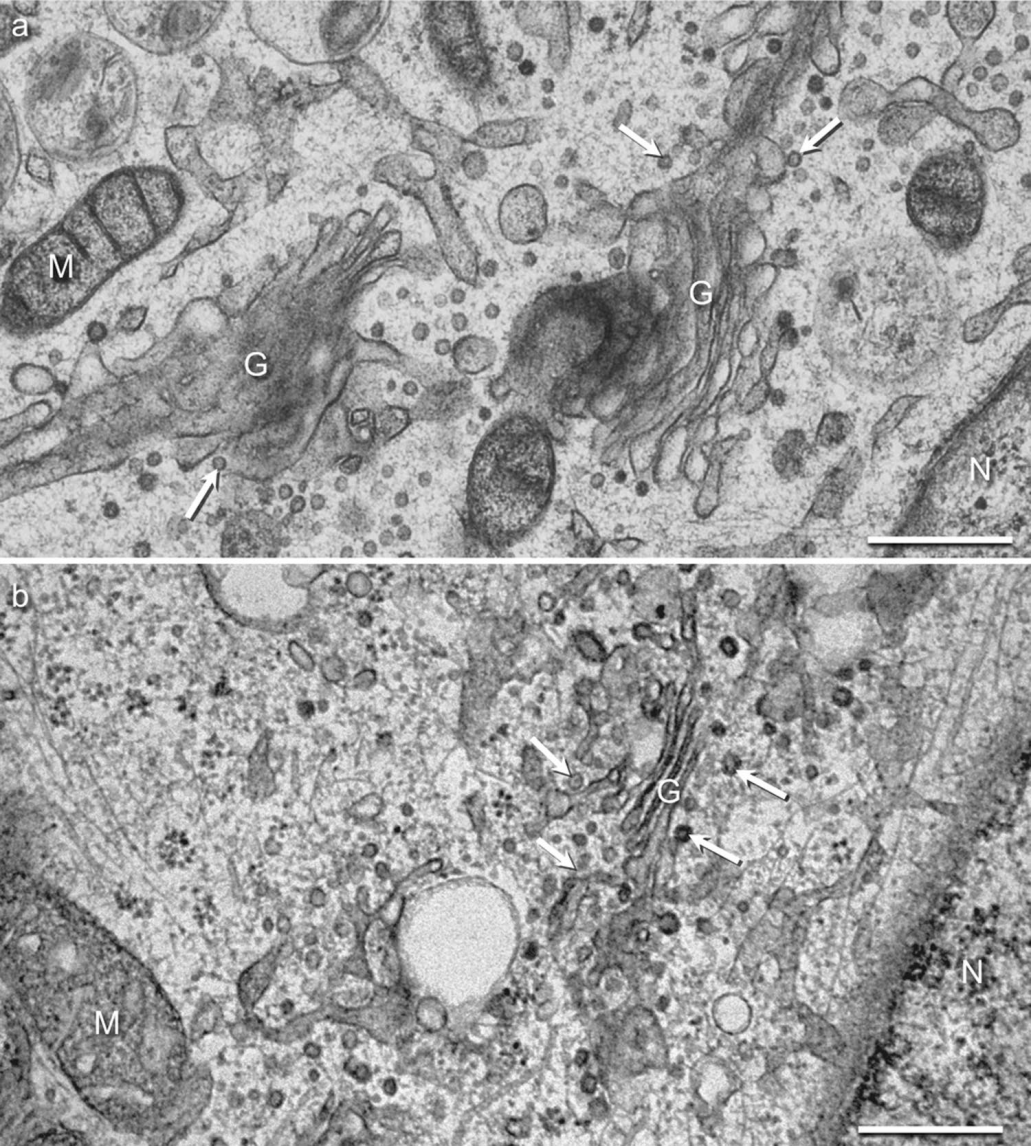
Structure of high-pressure-frozen uninfected glioblastoma cells without **a** and with **b** prefixation: Golgi cisternae (G) are well defined, and many Golgi-associated vesicles are visible (white arrows) in both samples. The mitochondria (M) are well preserved in **a** but showed swelling of the cristae after prefixation **b**. *N* nucleus. Bars 500 nm

Uninfected glioblastoma cells (cell line #12537 GB) had been high-pressure frozen from the native state without prefixation. They showed very prominent Golgi apparatus structures that took up a considerable amount of the cytoplasm, with well-defined cisternae as well as a high number of Golgi-associated vesicles (S1 Video in Supplementary). We consider these images and videos as the gold standard for ultrastructure of cultivated glioblastoma cells. For safety reasons, however, ZIKV-infected cells need to be prefixed with glutaraldehyde for the transport from the cell culture laboratory to our high-pressure freezer. To evaluate how prefixation may influence structure, we compared our natively frozen cells (Fig. 1a) with prefixed uninfected cells (Fig. 1b). The Golgi apparatus was clearly visible in both preparations. There was no considerable decrease of cisternae and Golgi-associated vesicles. Also, the relative position next to the nucleus remained unchanged. This indicates that chemical prefixation did not impair the visibility of the Golgi apparatus in this study. The structure of mitochondria, however, was clearly affected by prefixation, as we reported earlier (Walther et al. 2009).

### Structure of ZIKV-infected glioblastoma cells

**Fig. 2:**
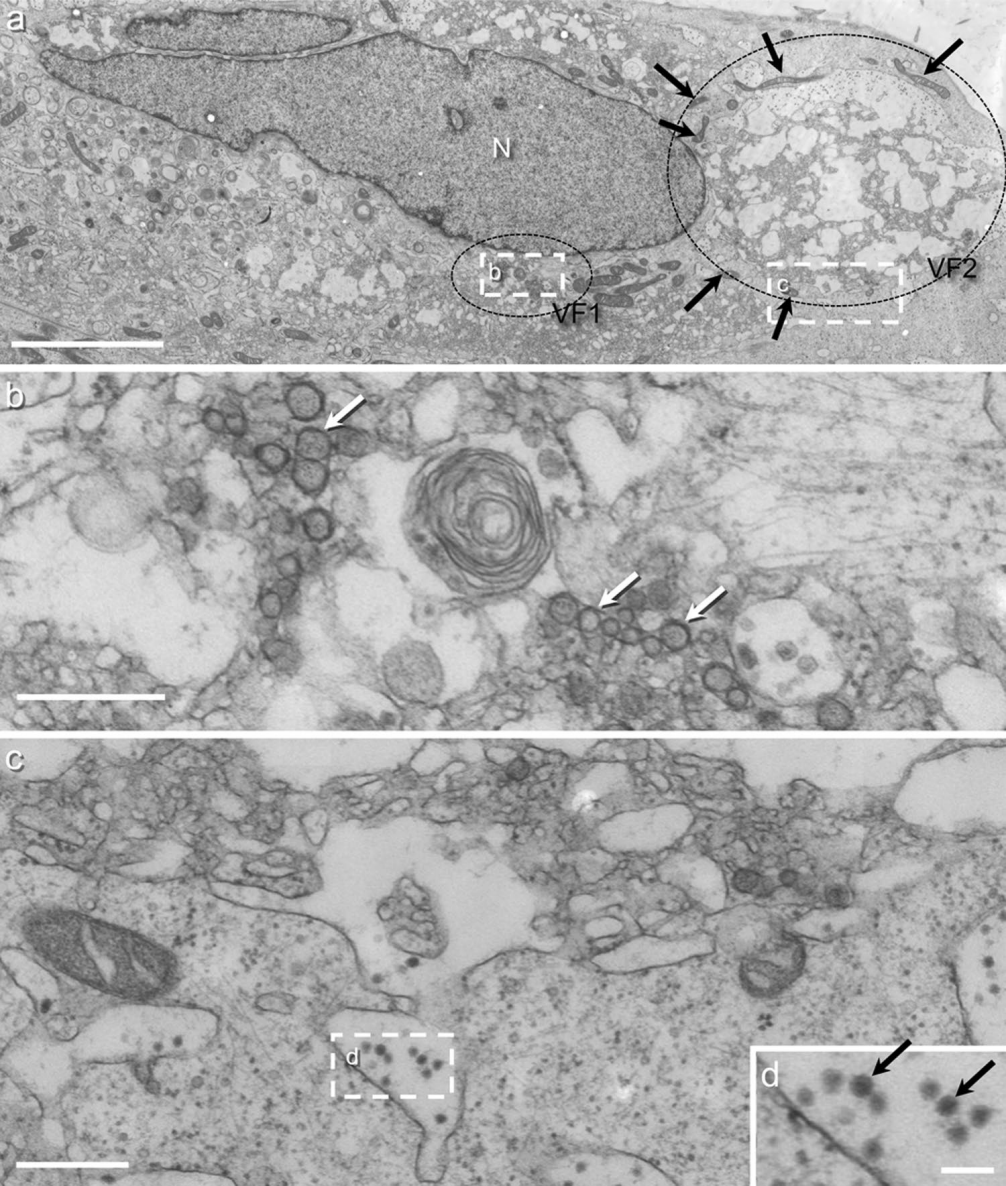
TEM composite image of a glioblastoma cell (cell line #12537 GB) infected with ZIKV and prefixed before high-pressure freezing. **a** Overview of two viral factories (encircled areas) visible in the cell, belonging to type 1 (VF1) and type 2 (VF2). The factories are surrounded by mitochondria (black arrows). **b** Higher magnification of the viral factories type 1. It contains many replication organelles (ROs) (some are marked with a white arrow) packed tightly in the surrounding ER. **c** Higher magnification of the border area of the viral factory type 2. The factory contains large, vacuole-like membranous compartments. Outside of the factories, the texture of the cytosol is like the texture in control cells, but canonical Golgi structures are absent. Bars: 10 µm (**a**), 500 nm (**b**, **c**), 100 nm (**d**)

Glioblastoma cells (line #12537 GB-GB) infected with ZIKV and prefixed before high-pressure freezing were imaged via TEM (Fig. 2). ZIKV infection led to a dramatic reorganization of the structures in the cytoplasm. Very large so-called viral factories occurred in the cytoplasm (encircled areas in Fig. 2a; enlarged in Fig. 2b, 2c). Outside of the factories, the texture of the cytosol was similar to control cells (Fig. 2c), consisting of densely packed particles, membranes, and filaments. Mitochondria were found surrounding the viral factories (black arrows in Fig. 2a), but were not located inside of them, suggesting either a very tight arrangement of organelles in the viral factory or a damaged cytoskeleton. The Golgi apparatus, the most prominent structure of uninfected cells, was missing in infected cells. Only putative residues of Golgi were found in some cells. Inside the viral factories, the cytoplasm appeared significantly altered. As various viral factories looked very different, we divided them into type 1 and type 2. The type shown in Fig. 2b is referred to as type 1, and the one in Fig. 2c as type 2. Both types consist of ER with largely changed morphology. Type 1 viral factories contain many densely packed replication organelles (ROs). These are luminal vesicles inside the ER. Viral factories of type 2 (Fig. 2c) contain fewer ROs but large, vacuole-like membranous compartments with ribosomes attached to them. These structures are formed by bloated ER, and in some areas they contain viral particles (Fig. 2d). Another example of a cell with viral factories type 1 and type 2 is shown in Supplementary Fig. S6.

**Fig. 3:**
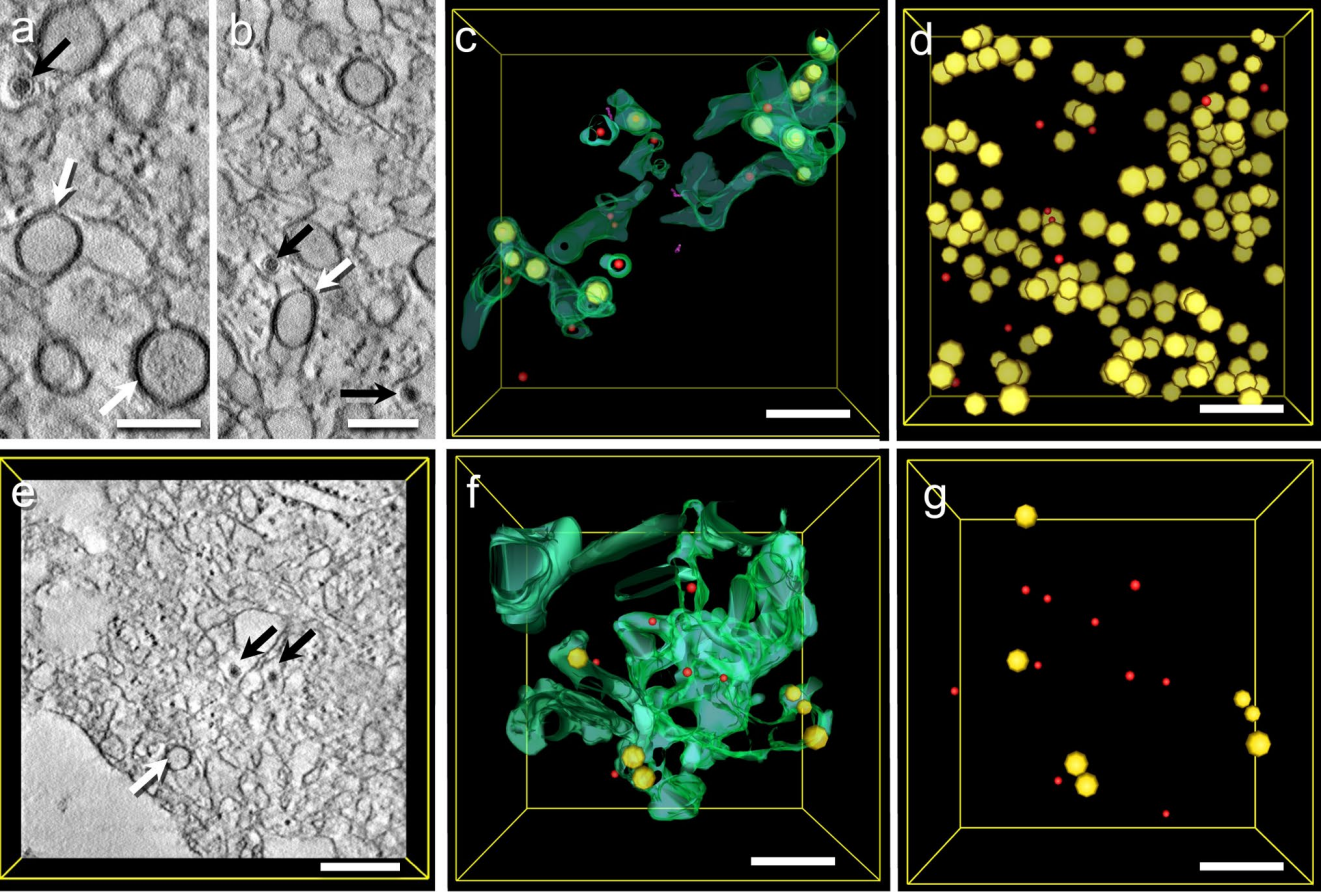
Tomograms of the two types of viral factories from cell line #12537 GB. **a**–**d** Tomogram of a type 1 viral factory in a ZIKV-infected cell (#12537 GB). The three-dimensional ER structure is segmented in green. The ER appears loose and independent of cytoskeletal arrangement as if a scaffold is missing. The most prominent structures are ROs (yellow). Often, a contrast-rich area between the inner layer of the RO and the ER can be identified (white arrows in the virtual sections **a** and **b**; yellow in the segmentations **c** and **d**). Virions were observed next to ROs in the ER lumen (black arrows in the virtual sections; red in the segmentations). In **c**, only a portion of the ER is segmented in green, and the viral particles and ROs in it are shown. In **d**, all ROs in the volume of the tomogram are shown. **e**–**g** Tomograms of a type 2 viral factory. The ER is swollen, and the total numbers of viral particles and ROs are considerably lower than in type 1 viral factories (compare **g** with **d**). Bars 100 nm (**a**, **b**), 250 nm (**c**–**g**)

The structures become clearer in tomograms acquired at higher magnifications (Fig. 3). A portion of a viral factory type 1 in a ZIKA-infected cell (#12537 GB) is recorded as a tomogram in Fig. 3a–d, with Fig. 3a and b showing portions of virtual sections, again showing ROs (white arrows) and viral particles (black arrows) surrounded by the ER membrane. Often, the area between the membrane of the ROs and the ER appears very dark. A portion of the three-dimensional ER structure is visualized in green in Fig. 3c containing some ROs (in yellow) and some viral particles (in red). The ER appears loose as if a scaffold is missing. The ROs are formed by luminal vesicles in the ER lumen. The membranes of ROs and ER are in very close proximity, but we observed neither open ROs nor membrane invagination stages of these ROs or any kind of membrane continuity between ER and ROs. Virions were observed next to ROs in the ER lumen. In Fig. 3c, only the ROs in the segmented portion of the ER are visualized; in Fig. 3d, however, all ROs and all virions in the tomogram are visible. A tomogram of a viral factory type 2 similar to Fig. 2c derived from cell line #12537 GB is shown in Fig. 3e–g. The segmentation shows ER that forms vacuolar structures. Individual virions (in red) reside inside those vacuoles. Occasionally, ribosomes remain connected with parts of the ER, but generally, they are missing. Interestingly, fewer ROs are found inside the ER of the type 2 viral factory (compare Fig. 3d with Fig. 3g).

**Fig. 4:**
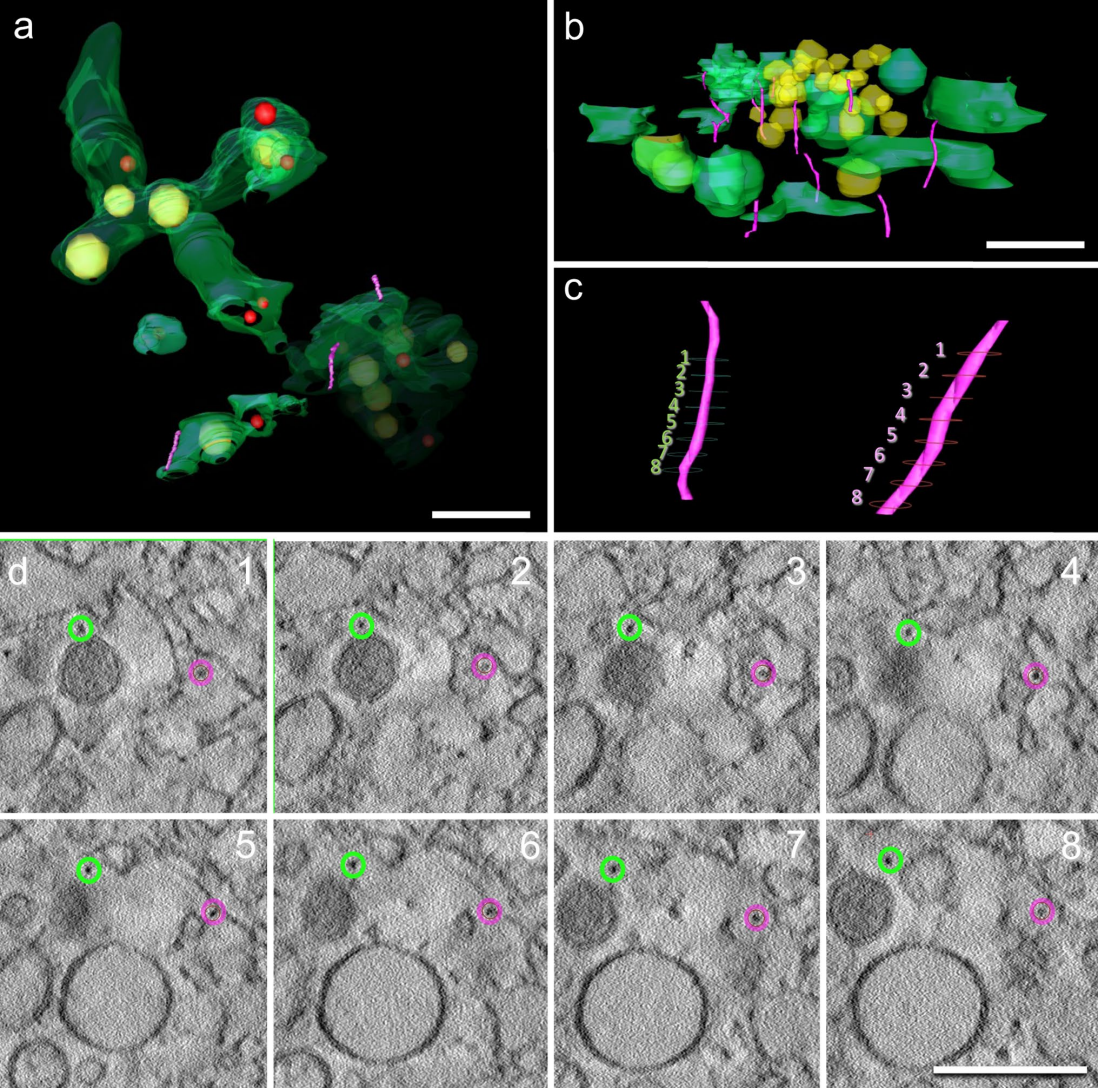
**a**–**d** Tomograms of a viral factory of type I derived from cell line #12537 GB. **a** is derived from a different cell than **b** and **c**. On three-dimensional reconstruction, we found many roughly 100-nm-long strings (violet) in close proximity to ROs and membrane compartments. Those strings occur as dark dots in the two-dimensional virtual sections (**d**). **d** shows two strings in eight different virtual sections (highlighted with green and violet circles), possibly resembling viral ribonucleoproteins (RNPs). The true 3D structure can only be recognized after segmentation (violet strings in **a**, **b**, and **c**) and becomes more clear in the Supplementary Video S4. Bars 200 nm

Figure 4 shows two tomograms of viral factories of type 1 (similar to Fig. 2b and Figure 4f) derived from cell line #12537 GB. On three-dimensional reconstruction, we found many roughly 100-nm-long strings near ROs and membrane compartments. Those strings occur as dark dots in the two-dimensional consecutive virtual sections (Fig. 4d 1–8). The true 3D structure can only be recognized after segmentation (pink strings in Fig. 4a, c).

### Cell line #15747 GB shows a different morphology

**Fig. 5:**
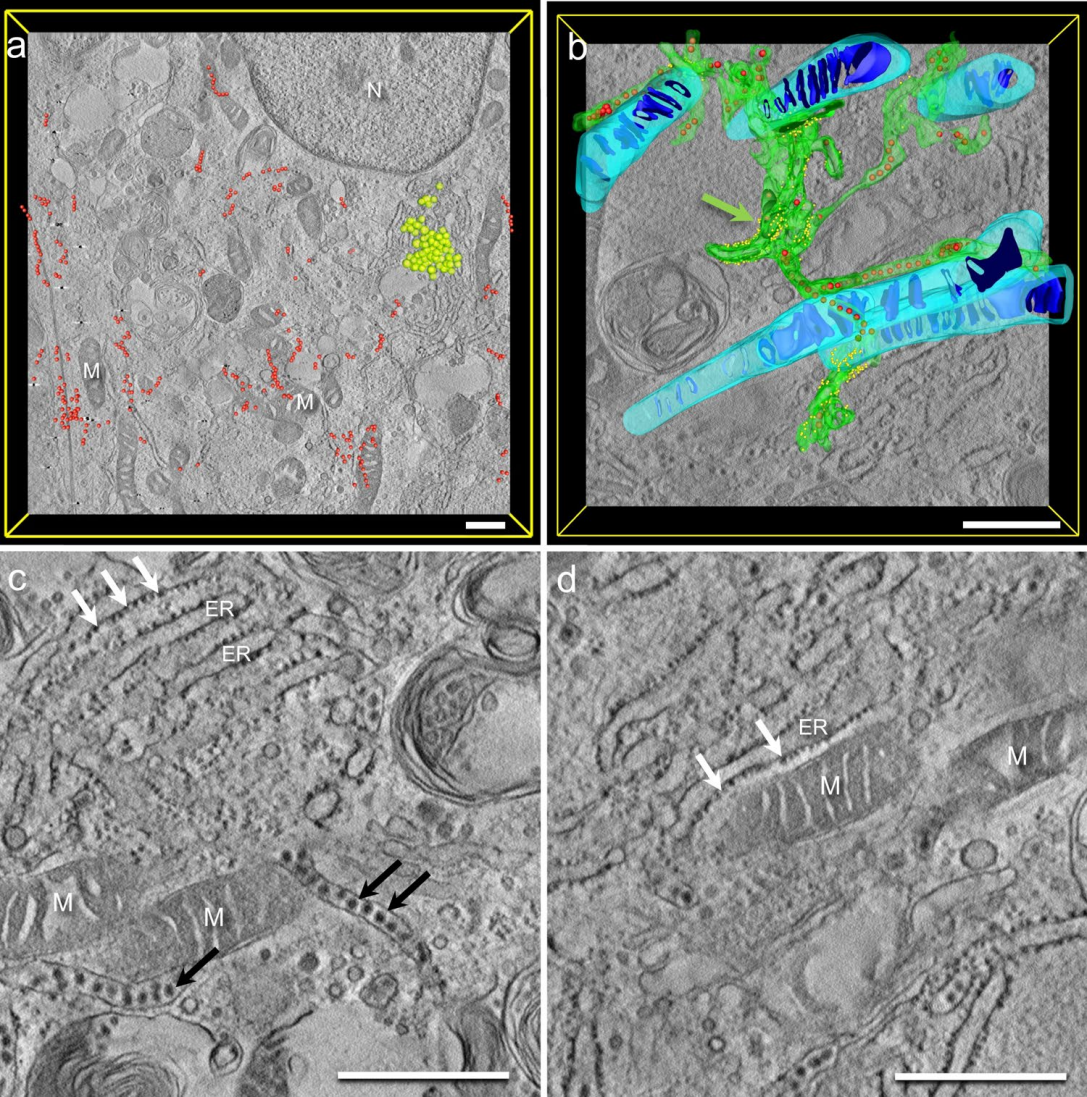
Tomograms of areas from cells of line #15747 GB. **a** The red virions in the altered ER are found at a significant distance from the viral factory with the yellow ROs. **b**–**d** Tomogram of another cell from cell line #15747 GB. The ER (green in segmentation) appears more tubular than in cell line #12537 GB. Virions (segmented in red, black arrows in the virtual sections) are arranged in pod-like ER structures devoid of ribosomes. Ribosomes (segmented in yellow or labeled with white arrows can be found in other areas of the same ER lumen). Continuation of these cisternae containing viral particles with rough ER could be demonstrated in the segmentation (**b**). Intact as well as virus-altered ER was found in close vicinity to mitochondria (white arrows in **d**). In contrast to #12537 GB, altered ER retained an organized three-dimensional structure with distinct 3D junctions (green arrow) that is especially well visible in video S5. All ER structures featured in the tomogram of **b**–**d** are connected with each other. Bars 500 nm

In cell line #15747 GB, virions (red) were found at a considerable distance from ROs (yellow), as visible in Fig. 5a. ROs also were found to have a significantly larger diameter compared with ROs in #12537 GB. An average diameter of 200.79 nm was determined for 53 ROs from a tomogram of an infected cell from #15747-GB (standard error of the mean, 4.94 nm). In comparison, for #12537 GB, the average diameter was 127.52 nm for 139 ROs from one tomogram (standard error of the mean, 1.27).

Cell line #15747 GB showed a more tubular ER (green), with two domains, one containing many tightly packed virions arranged in pod-like structures and one of an appearance similar to uninfected cells with ribosomes still attached (Fig. 5b–d). ER-areas with viral particles were devoid of ribosomes. Continuation of the cisternae containing viral particles with rough ER could be demonstrated in the segmentation (Fig. 5b). Notably, ER containing virions was mainly found in areas with intact cytoplasm as described above. Intact as well as virus-altered ER was found in close vicinity of mitochondria (white arrows in Fig. 5d). The close proximity of altered ER and mitochondria is an outstanding feature of #15747 GB, since mitochondria were not found within the viral factory of #12537 GB but rather located around it. Also, in contrast to #12537 GB, altered ER retained an organized three-dimensional structure with distinct 3D junctions (see Video Supplement S3). All ER structures in the tomograms in Fig. 5c and d are connected with each other.

### Sandfly fever Turkey virus

**Fig. 6:**
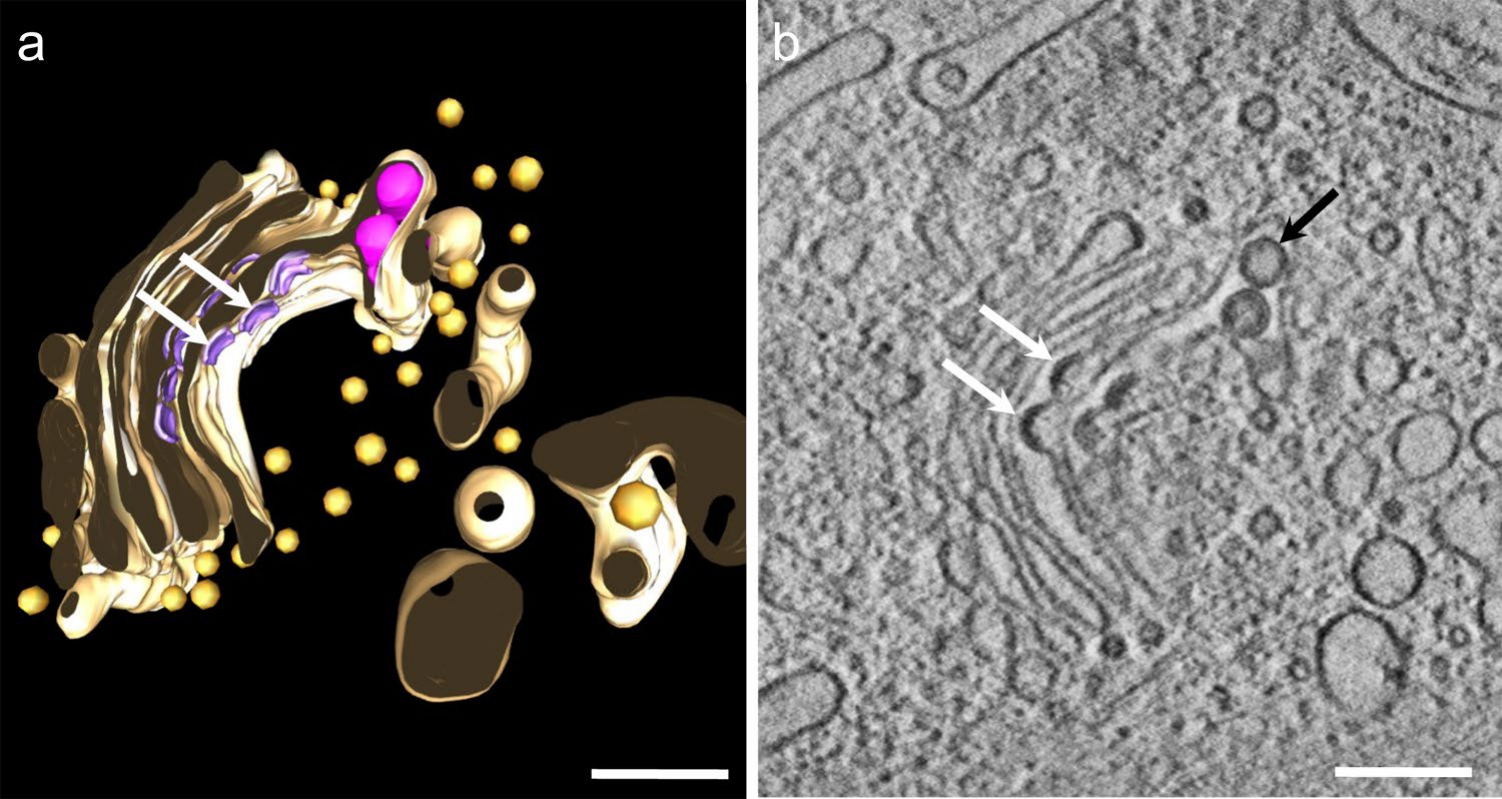
Tomogram of a cell infected with sandfly fever Turkey virus, a phlebovirus, where the viral capsids are formed in the Golgi apparatus (arrows point to budding virions), in contrast to ZIKV, where capsids are formed in the ER. Golgi cisternae colored in brown, phlebovirus capsids in violet, Golgi-related vesicles in orange. Bars 200 nm

Figure 6a shows the segmentation of a tomogram of a cell infected with sandfly fever Turkey virus. In Fig. 6b, a slice through the tomogram from Fig. 6a shows the forming of the viral capsids in the Golgi apparatus. The reconstruction shows intact canonical Golgi structures with well-defined cisternae and COP vesicles, comparable to the data shown in Fig. 1. Virions are found in the lumen of the Golgi. Invaginations of the Golgi are budding virions.

## Discussion

Natively frozen samples (without prefixation) are the gold standard for ultrastructural observations (Wolff et al. 2020b; Mielanczyk et al. 2014). Unfortunately, for safety reasons, ZIKV-infected cells need to be chemically prefixed, which could result in structural alterations. However, as the comparison of the structure of prefixed and non-prefixed controls shows, the Golgi apparatus can still be identified after chemical fixation (Fig. 1). In ZIKV-infected glioblastoma cells, viral factories consisting of enlarged ER are the main site of virion formation. Canonical Golgi structures, however, were absent, and only withered remains could be observed in the electron microscope. We believe the loss of Golgi structures to be a feature of ZIKV infection since it was only observed in infected cells. This agrees with the findings of Cortese et al. (2017), where fluorescence microscopy revealed the Golgi-specific GM130 signal of ZIKV infected Huh7 cells to be significantly altered in comparison with mock infected cells. Fluorescence microscopy imaging shown by Cortese et al. shows mock infected cells with a well-defined, cisternae-shaped Golgi apparatus comparable to the ultrastructural data shown in Fig. 1. For ZIKV-infected cells, the GM130 signal was found in a larger area around the nucleus and no regular shape could be identified. This correlates with our data, where no classical Golgi stacks were visible in ZIKV-infected cells. Previous investigations, however, proved Golgi proteins to be involved in ZIKV virion maturation (Sager et al. 2018). Cortese et al. (2017) also found the signal of the Golgi membrane and trans-Golgi network to be translocated to the area containing a marker for dsRNA, which is only found as intermediate in the viral replication cycle. Together with the absence of Golgi structures, this could hint that in ZIKV-infected cells the Golgi has fused with the ER and thus has become a part of the viral factory. In contradiction to our data, Rossignol et al. (2017) show ZIKV-infected Vero E6 cells having a well-defined Golgi apparatus. This could possibly be explained by the usage of a different virus strain and a different cell line.

We did not observe physiological Golgi structures in our ZIKV-infected cells, which leads us to the conclusion that in ZIKV infection the Golgi proteins and membranes are needed solely for virion maturation rather than cellular transport. In phlebovirus, however, the Golgi apparatus is the main site of virion formation. As visible in Fig. 6, virions bud into the lumen of the Golgi and are transported to the cell surface by COP-vesicles. Therefore, phleboviruses utilize the physiological transport function of the Golgi apparatus, and the Golgi retains its physiological structure.

We observed two types of viral factories in the Zika-infected cells of cell line #12537 GB. Type 1 has a higher density of ROs and viral particles than type 2. In type 2 viral factories, the ER is swollen and forms large vacuoles. Due to the ultrastructural changes, we assume that type 1 is an earlier stage and type 2 is a later state of the viral factory, since type 1 is a more ordered and type 2 a more chaotic structure. We consider the structure of type 1 viral factories comparable to other published data of ZIKV infection, like the findings of Hanners et al. (2016), Offerdahl et al. (2017), and Cortese et al. (2017).

With the help of STEM tomography of relatively thick sections (600 nm), we could show luminal vesicles within the ER lumen of infected cells (Fig. 3) that correspond to the concept of replication organelles (ROs). These structures have already been shown in cells infected with Zika (Cortese et al. 2017) or dengue (Welsch et al. 2009). In 2D representations (e.g., Fig. 3b, these structures look like vesicles with two surrounding membranes (“double membrane vesicles”), and only after 3D segmentation does it become obvious that the outer membrane is in fact the ER and only partially in touch with the luminal vesicle.

We did not observe pores in the replication organelles (ROs) of Zika-infected cells. This contrasts with the data of Welsch et al. (2009) clearly showing pores in ROs of dengue-virus-infected cells that seem to occur due to invaginations of ER membranes that then do not pinch off (incomplete fission). We observed neither open ROs nor membrane invagination stages of these ROs or any kind of membrane continuity between ER and ROs. We find this an interesting morphological difference to the dengue virus shown in the Welsch paper, and we believe that this indeed represents a different biological state and not a preparation artifact since our preparation is similar to the preparation protocol in Welsch et al. (2009).

Whereas these differences can probably be explained by the different viral species, dengue and Zika, it is more difficult to explain why we could not see the pores in the ROs reported by Cortese et al. (2017) for Zika virus. In Fig. 4D of Cortese et al. (2017), where the authors claim to see a pore, the inner and the outer membrane of the RO do not show a connection; so, there is no indication of a pore formed by an incomplete invagination as shown for dengue virus by Welsch et al. (2009). Hence, one might suspect that the structures observed by Cortese et al. (2017) are different from the pores observed in dengue-infected cells.

The ROs in our data share major structural similarities with the double membrane vesicles (DMVs) of severe acute respiratory syndrome coronavirus 2 (SARS-CoV-2) reconstructed from Cryo-EM (Wolff et al. 2020b). We assume that these DMVs are intraluminal vesicles surrounded by the ER membrane, similar to the ROs in Zika virus. However, we did not find any early stages of ROs (e.g., incomplete fission) in our Zika-infected cells. Therefore, it remains open to us how the ROs are formed. It seems likely that they are formed by invagination of the ER membrane as described by Welsch et al. (2009) and Cortese et al. (2017) or by mechanisms like de novo formation of autophagic membranes. However, we could not find structural evidence for either pathway in our data.

As mentioned in the introduction, Wolff et al. (2020b) were able to show pores formed by proteins to transfer the replicated RNA into the newly forming virions at the ER membrane similar to nuclear pores. We estimate that such a pore would not be visible on our resin-embedded samples. Given that we found no pore resulting from incomplete membrane fission, it would seem likely that ZIKV uses a similar strategy. However, the smaller genome of ZIKV may not have enough capacity for the proteins of a nuclear-pore-like complex.

We observed strings with a length of roughly 100 nm in viral factories of cell line #12537 GB (Fig. 4). We believe that these strings represent ribonucleoprotein complexes (RNPs) on their way from the replication site in the ROs through the cytosol to the newly budding virions in the ER. As mentioned above, such structures have been predicted but thus far have only been observed in ROs by Klein et al. (2020) and not in the cytosol like suggested by Wolff et al. (2020b), Fig. 4. This could be for technical reasons: in contrast to cryo-TEM as used by Wolff et al. (2020b), our samples are stained with heavy metals during freeze substitution to provide sufficient contrast. This may enhance the contrast not only of membranes and proteins in the sample but also of structures such as RNPs. In addition, we used thicker sections (600 nm compared with 100 nm in cryo-TEM), which may may increase the probability of finding whole ROs in one tomogram. All RNPs that we observed are almost perpendicular to the *x*–*y* plane of the section. Most likely, RNPs arranged parallel to the *x*–*y* plane are not visible with our approach, since EM tomography is an anisotropic imaging mode and the resolution is better in *x* and *y* than in *z*.

Cell line #15747 GB shows a different morphology compared with #12537 GB, despite having been infected with the same virus strain at the same MOI for the same amount of time. In addition to a small viral factory containing ROs, cells of #15747 GB have a slightly altered ER that is filled with virions and closely connected to mitochondria. We assume that virions are transported from the viral factories to their current positions and possibly remain trapped in the ER lumen. This would be the case if the viral maturation process could not be completed. Interestingly, ROs in #15747 GB have a significantly larger diameter than in #12537 GB. However, the reason for this and possible effects on virus replication remain unclear.

Altered ER was found close to mitochondria (Fig. 5d), thus forming structures resembling mitochondria-associated membranes (MAMs). Notably, flavivirus infection like ZIKV or dengue virus is known to disrupt mitochondria-associated membranes (Chatel-Chaix et al. 2016; Neufeldt et al. 2018). The intact MAMs found in #15747 GB could hint at an impaired replication cycle. The fact that the ER appears loose in #12537 GB and more defined in #15747 GB could indicate that the cytoskeleton in the viral factory of #15747 GB is more stable compared with #12537 GB. Altogether, ZIKV infection of cell line #15747 GB significantly differs from #12537 GB and other published cell lines infected with Zika. However, the exact cause of the different morphology and the consequences on virion production and cell lysis remain to be examined, and we cannot exclude that the differences are a result of different replication kinetics.

## Conclusions

– Golgi structures are still clearly visible after chemical prefixation.

– Golgi structures in infected cells are either absent or withered.

– Cytoplasm outside of the viral factory appears unaltered.

– Two types of viral factories can be distinguished. Both types can be found in the same cell.

– Assembled virions and VF are present in #15747 GB but situated at different sites in the cell. This could hint at a dysfunctional transport of virions in #15747 GB, resulting in virions stuck in the ER lumen. On the other hand, we cannot exclude that the differences are a result of different replication kinetics.

– We found strings near ROs that could represent RNPs on their way to the viral particles. Such structures have so far not yet been shown in the cytoplasm but in the lumen of ROs (Klein et al. 2020).

- Structural alterations in the host cell caused by ZIKV, where viral particles are assembled in the ER, are very different from structural alterations caused by the phlebovirus sandfly fever Turkey virus (a minus-strand RNA virus), where viral particles are assembled in the Golgi apparatus.

## Supplementary Information

Below is the link to the electronic supplementary material.

S1: Video “S1 Golgi of uninfected glioblastoma cell” corresponds with Fig. 1a and shows the 3 D structure of a Golgi apparatus of an uninfected and not prefixed glioblastoma cell of cell line #12537-GB (MP4 34112 kb)

S2: Portion of the composite image of the infected cells of cell line #15537-GB, shown in Fig. 2. The image is very large, and one can zoom in to see the viral particles with good resolution. Viral factories of type 1 and type 2 are encircled. Caution: Since the image is very large (15k x 6k pixels) it may not open with all computers. In our hands it opens well with “imageJ” (JPG 24635 kb)

S3: Portion of a composite image of infected cells of cell line #15747-GB. The encircled area is a viral factory surrounded by mitochondria. Many viral particles in pod-like ER structures (arrows) can be found by zooming in, in large distance of the viral factory in (JPG 18378 kb)

S4: Video S4 RNPs in a viral factory” corresponds with Fig. 4 and shows a portion of a type 1 viral factory. The red circles circumvent strings that occur as dark dots in the individual virtual sections. Since the dots occur in consecutive sections, the 3 D structure is a string (MP4 53105 kb)

S5: Video “S5 pod like ER structures with virions” corresponds with Fig. 5 and shows a portion of a viral factory from cells of line #15747-GB. The viral particles (red in the segmentation) are arranged in pod like arrangements of the ER, that is connected with areas of the ER that still have ribosomes (yellow) attached (MP4 35776 kb)

S6: Video “S6 phlebovirus virion genesis in Golgi” corresponds with Fig. 6 and shows viral capsids formed in the Golgi apparatus (MP4 15345 kb)
